# Outcomes of general anesthesia vs. local anesthesia with monitored anesthesia care for elective umbilical hernia repair in adults: a propensity score- matched analysis

**DOI:** 10.1007/s10029-026-03704-9

**Published:** 2026-05-07

**Authors:** Sergio Huerta, Crystal Phung, Jared McAllister, Sri Tummala, Shirling Tsai

**Affiliations:** 1 Department of Surgery, VA North Texas Health Care System, University of Texas Southwestern Medical Center, 4500 S. Lancaster Road (112), Dallas, Texas 75216 USA; 2 Texas A&M College of Medicine, Dallas, Texas USA

**Keywords:** Umbilical hernia, Local anesthesia, Monitored anesthesia care, General anesthesia, Veterans, Hernia repair

## Abstract

**Introduction:**

There are no randomized controlled trials comparing general anesthesia (GA) to local anesthesia with monitored anesthesia care (LA + MAC) for adult patients undergoing elective umbilical hernia repair (UHR). We hypothesized that LA + MAC would be associated with fewer postoperative complications without increasing recurrence.

**Methods:**

A retrospective analysis of a prospectively maintained database was performed, including consecutive Veteran patients undergoing elective open primary UHR at a single institution between August 2005 and June 2025. Patients undergoing emergent repair, laparoscopic repair, incisional hernia repair, or epigastric hernia repair were excluded. Primary outcomes were recurrence, 30-day postoperative complications, and operative room times in patients receiving GA vs. LA + MAC. Variables significant on univariable analysis were included in a propensity score–matched analysis.

**Results:**

A total of 602 patients underwent UHR with GA (*n* = 427) or LA + MAC (*n* = 175). PSMA yielded 143 patients in the GA and 175 patients in the LA + MAC group. In the unmatched cohort, recurrence was higher after GA than LA + MAC (4.7% vs. 1.1%, *p* < 0.01), but this difference was not significant after matching (2.8% vs. 1.1%, *p* = 0.30). Overall, 58 complications occurred (54 GA vs. 4 LA + MAC). In the unmatched cohort, complication rates were higher with GA (12.6% vs. 2.3%, *p* < 0.01), and this difference persisted after matching (9.8% vs. 2.3%, *p* < 0.01). Operative room time was modestly shorter with LA + MAC (mean difference = 5.3 min).

**Conclusions:**

LA + MAC was associated with significantly fewer postoperative complications and modestly shorter operative time, without increased recurrence. These findings support consideration of LA + MAC for elective open UHR.

## Introduction

Umbilical hernias (UH) are commonly encountered in the practice of a general surgeon. 10% of all abdominal hernias are UHs, with a prevalence of 2% in adults [1]. In 2012, an estimated 180,700 Americans underwent umbilical hernia repair (UHR) [2]. Most adult UHs are associated with increased intra-abdominal pressure, including obesity, pregnancy, and ascites [1–3]. Small, asymptomatic UHs might be observed depending on the surgical risk of the patient. When patients develop symptoms, are uncomfortable with a UH, or desire repair, multiple surgical options are available, including open tissue repair, open mesh repair, laparoscopic repair, and robotic UHR [1, 2]. The current best approach for UHR depends on the patient population, the hospital’s resources, the surgeon’s expertise, and patient preferences. Globally, most UHs are repaired via the open approach [1].

A key advantage of open UHR is that it can be done under local and monitored anesthesia care (LA + MAC) instead of general anesthesia (GA). Potential advantages of LA + MAC over GA include decreased intraoperative time [4], reduced postoperative pain [4], earlier ambulation, decreased length of hospital stay [5], fewer postoperative complications (e.g., less urinary retention) [6], and potential cardiac complications in high-risk patient populations [7]. Additionally, LA + MAC can lead to a decrease in the cost associated with the operation compared to GA [4, 6, 8, 9]. While there is current level I evidence for the repair of inguinal hernias under LA + MAC [7], for UH, this evidence is less clear. There are no randomized controlled trials (RCTs) comparing UHR under LA + MAC vs. GA [8]. Accordingly, the European Hernia Society and the American Hernia Society stipulate that there is no strong evidence to demonstrate that LA + MAC is superior to GA for UHR [10]. The best current evidence for UHR under LA + MAC emanates from two systematic reviews, which have shown only the safety and feasibility of this approach for the repair of UHs, especially in elderly, frail individuals [8, 11]. A prior study at our institution (*n* = 53) found LA + MAC to be a feasible approach in Veteran patients submitting to open UHR [12].

The present study was conducted to evaluate outcomes in Veteran patients undergoing UHR under LA + MAC versus GA using a propensity-score-matched analysis (PSMA). We hypothesize that after correcting for major variables, LA + MAC would result in better outcomes compared to GA for Veteran patients undergoing open UHR.

## Methods

### Study design

The present analysis is a single-institution, single-surgeon retrospective review of a prospectively maintained database of all consecutive Veteran patients presenting to the VA North Texas Health Care System (VANTHCS) for elective open umbilical hernia repair (UHR) between August 2005 and June 2025. Approval of this study was obtained from the Institutional Review Board (IRB) of VANTHCS. Because all data analyzed were retrospective and de-identified, the requirement for informed consent was waived by the VANTHCS IRB.

### Eligibility criteria

A prospectively maintained database of the primary surgeon at the VA North Texas Health Care System (VANTHCS) was queried to identify all consecutive adult Veteran patients undergoing UHR between August 2005 and June 2025. Inclusion was limited to patients undergoing elective open primary umbilical hernia repair (UHR). Patients undergoing emergent repair, laparoscopic repair, incisional hernia repair, or primary epigastric hernia repair were excluded. After applying these criteria, the final cohort consisted of 602 patients .

In April 2017, a local anesthesia with monitored anesthesia care (LA + MAC) program was implemented. Thereafter, all patients were offered LA + MAC, with patient refusal or allergy to local anesthetic agents as the only contraindications. Patients not undergoing LA + MAC received general anesthesia (GA).

**Fig. 1:**
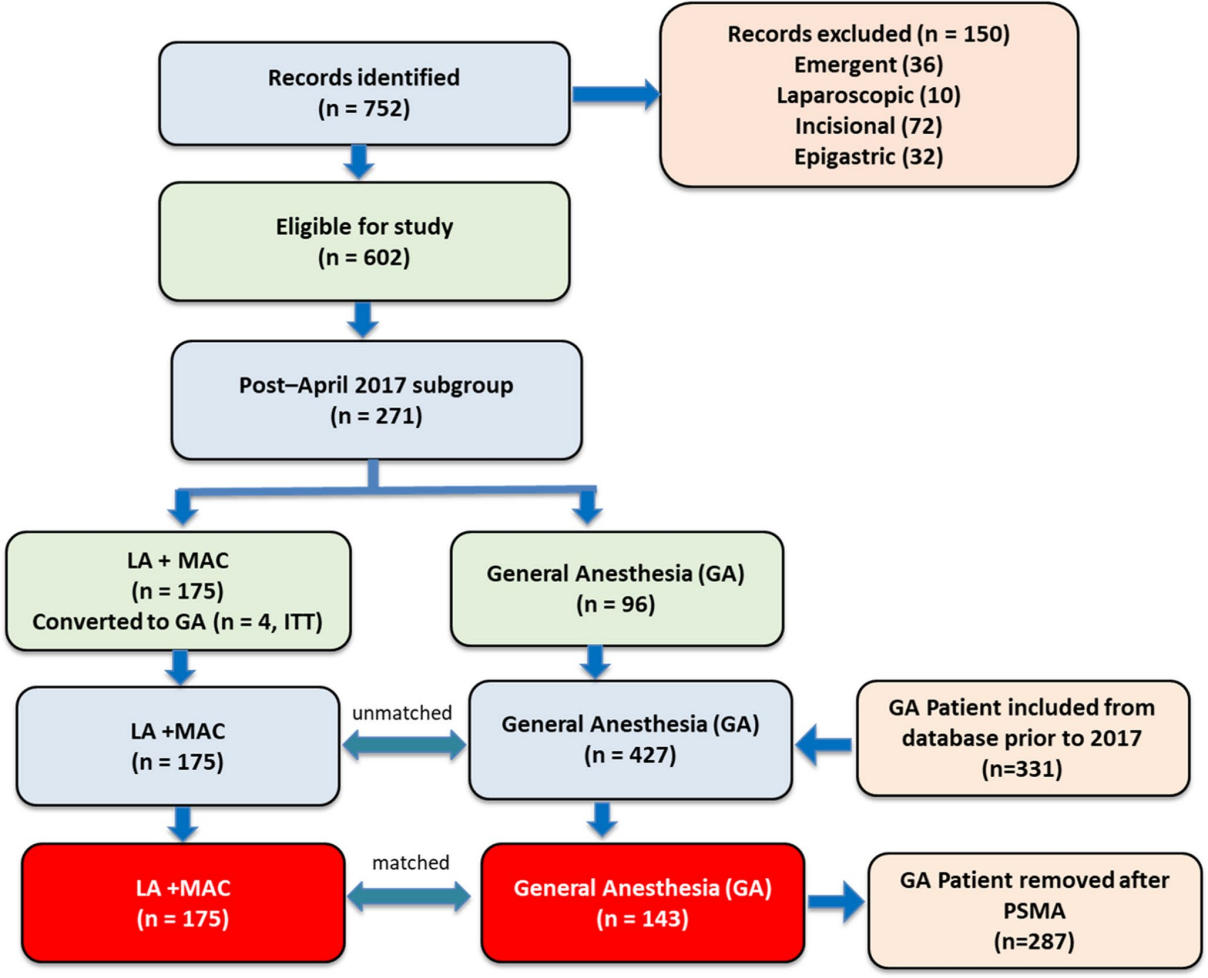
Flow diagram illustrating patient selection, exclusions, and cohort development for the study.The figure details the application of inclusion and exclusion criteria, subgroup stratification, and final cohort formation following propensity score–matched analysis. LA = local anesthesia; MAC = monitored anesthesia care; ITT = intention-to-treat; GA = general anesthesia; PSMA = propensity score–matched analysis

Among patients treated after implementation (*n* = 271), 175 (64.6%) underwent LA + MAC. Patients requiring intraoperative conversion from LA + MAC to GA were analyzed using an intention-to-treat approach (Fig. 1).

### Outcomes

The primary endpoints were hernia recurrence and overall postoperative complications within 30 days of surgery, comparing patients undergoing repair under general anesthesia (GA) versus local anesthesia with monitored anesthesia care (LA + MAC). The average follow up for hernia recurrence was (9.5 ± 5.4 years). These outcomes were analyzed in both the unmatched cohort and the propensity score–matched cohort. Another primary endpoint included operative room time metrics (total operative time and room utilization time).

Secondary endpoints included the incidence of specific postoperative complications, including surgical site infection (SSI), seroma, hematoma, and cardiopulmonary events, compared between groups. SSI was defined according to Centers for Disease Control and Prevention (CDC) criteria. Seroma and hematoma were defined as clinically evident fluid collections requiring observation or intervention. Cardiopulmonary complications included events such as pneumonia, respiratory failure, myocardial infarction, and arrhythmias occurring within the 30-day postoperative period.

### Data collection

Demographic data were collected prospectively. After each procedure, the primary author entered patient age, race, sex, body mass index (BMI), American Society of Anesthesiologists (ASA) classification, and anesthesia type into a standardized database. Medical comorbidities recorded included hypertension, diabetes mellitus, chronic obstructive pulmonary disease, cardiovascular disease (including coronary artery disease, arrhythmias, and valvular disease), liver disease, ascites, and chronic anticoagulation use. Preoperative serum albumin levels were recorded. Smoking status was categorized as current (within 12 weeks of the operation), former, or never smoker.

Hernia-specific variables were recorded immediately after each case and included hernia size and the presence of chronic incarceration. Duration of hernia symptoms was obtained from history and physical examination. Operating room times were recorded by the circulating nurse and included time from patient entry into the operating room to incision, skin-to-skin operative time, and time from skin closure to exit from the operating room.

### Identification of recurrence and postoperative complications

We have previously reported methods of identification of postoperative complications and recurrence [12]. Briefly, recurrence was identified through three complementary mechanisms. (1) Clinical examination during postoperative follow-up or evaluation of patient-reported symptoms such as pain, discomfort, or development of a new bulge. Computed tomography imaging was obtained when clinically indicated. (2) Comprehensive review of all electronic medical records for documentation of suspected recurrence by any treating provider. (3) An independent review of all available computed tomography imaging obtained for any indication during follow-up by the primary author, in conjunction with radiology reports.

Postoperative complications were identified through review of the electronic medical record during the 30-day postoperative period and beyond. Patients evaluated in clinic, emergency department, or by their primary care physician were entered into the prospectively maintained umbilical hernia database. Each complication was counted once per patient, as documented in the electronic medical record. When multiple related complications were recorded, only the highest-severity event was included (e.g., surgical site infection rather than cellulitis; ileus rather than nausea; constipation rather than nausea). In each case, the highest level of complication per patient was reported.

### Operative technique

All umbilical hernia repairs were performed by the same surgeon using a standardized open surgical technique that has been described previously [1–3].

#### Anesthesia

##### General anesthesia

GA was administered by anesthesiology staff using either an endotracheal tube or a laryngeal mask airway at their discretion.

##### **Local anesthesia**

**Fig. 2:**
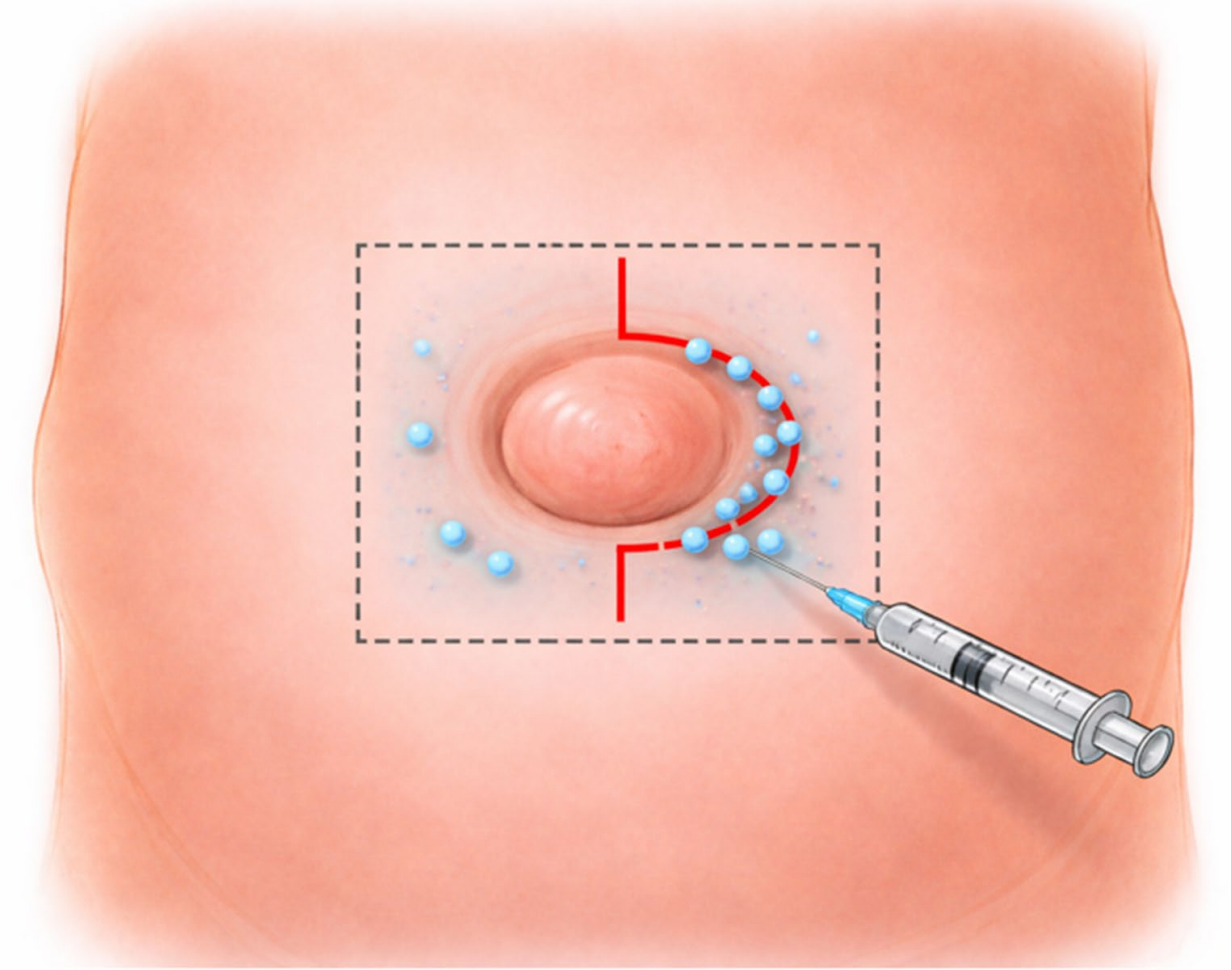
Technique for application of local anesthesia and initial incision for open umbilical hernia repair. The red curvilinear line represents the typical incision, extending approximately 1–3 cm above and below the umbilicus along its left lateral aspect. This line also marks the initial site for local anesthetic infiltration. The dotted square delineates the broader field of local anesthetic administration to ensure adequate regional coverage prior to incision

For patients undergoing LA + MAC, local anesthesia was administered by the operating surgeon as a field block around the umbilicus (Fig. 2). Local anesthetic consisted of 1% lidocaine with epinephrine (1:100,000) combined with sodium bicarbonate (2.5 mEq per 5 mL). An initial volume of 30 mL was administered, with additional anesthetic provided as needed to ensure adequate patient comfort. We have described this approach elsewhere [1].

##### Monitored anesthesia care

Sedation most commonly included propofol administered as a continuous infusion, often supplemented with small doses of midazolam, fentanyl, ketamine, or additional propofol boluses as clinically indicated. Sedative selection and dosing were individualized based on surgical stimulus, patient comorbidities, and mental status. Patients remained spontaneously breathing, easily arousable, and hemodynamically stable, which allowed bypass of the post-anesthesia care unit. We have described this approach previously [1].

#### Surgical technique

A vertical curvilinear incision is made approximately 1–3 cm above and below the umbilicus, depending on the size of the hernia. The incision is carried along the left lateral aspect of the umbilicus (Fig. 2). Dissection is continued through the subcutaneous tissue using electrocautery. The umbilicus is carefully dissected free from its attachment to the underlying umbilical ring, taking care to avoid buttonholing the skin.

The neck of the umbilical hernia is identified and measured. The surrounding fascia is dissected free from the hernia sac. In cases where the hernia contents are small, reduction is performed through the defect. The fascial edges are circumferentially cleared using a right-angle clamp to facilitate exposure.

If a chronically incarcerated component cannot be reduced through the hernia neck, the hernia sac is opened and its contents are inspected. When feasible, reduction is performed following release of adhesions. If only omentum is present and cannot be reduced, partial omentectomy is performed using an energy device (LigaSure™ or equivalent). After complete reduction of contents, the fascial edges are inspected to ensure a circumferential clearance of approximately 2–3 cm and to confirm the absence of adherent intra-abdominal structures.

In our practice, most umbilical hernias are repaired using a primary tissue repair technique, irrespective of hernia size or patient body mass index, as previously described [1, 3–5]. The fascial defect is closed using interrupted 1 − 0 polydioxanone (PDS) sutures placed in a vertical figure-of-eight fashion. Typically, two to four sutures are sufficient depending on defect size.

The umbilicus is then reattached to the underlying fascia using a 2 − 0 polyglactin (Vicryl) suture. The subcutaneous tissue is approximated with interrupted 3 − 0 Vicryl sutures, and the skin is closed with a running 4 − 0 Monocryl suture. Skin adhesive (Dermabond™) is applied to the incision for final closure.

In cases where the fascia is assessed to be weak, or in the setting of recurrent hernias, a small piece of dual-sided mesh is placed in an underlay position. The mesh is secured with interrupted 0-Ethibond sutures. The fascia was then closed over the mesh using 0-PDS suture.

### Statistical analysis

All data were entered into a Microsoft Excel spreadsheet and de-identified prior to analysis. Patients were stratified into two groups based on anesthesia type: GA and LA + MAC. Continuous variables are presented as mean ± standard deviation and were compared using Student’s t-test. Categorical variables were compared using *Chi-Square* or Fisher’s exact tests, as appropriate. Statistical significance was defined as a two-sided *p*-value less than 0.05. All statistical analyses were conducted using IBM SPSS ver. Version 30.0.0.0 (172) (IBM Corp., Armonk, NY, USA).

Variables demonstrating statistical significance in univariable analysis were included in a propensity score matching model. Propensity score matching was performed in a 1:1 fashion using a fuzzy matching extension with nearest neighbor matching and a caliper width of 0.1. Variables included in the matching model were BMI (obese ≥ 30 kg/m² vs. non-obese), past and current smoking history, diabetes mellitus, recurrent hernia repair, serum albumin level (≤ 3.4 vs. >3.4 mg/dL), repair type (primary vs. mesh), hernia size category (0.1–1.0, 1.1–2.0, 2.1–3.0, and > 3.1 cm), and operative time dichotomized at the cohort median of 41 min. All statistical analyses were performed using SPSS statistical software. Missing data were minimal and handled using standard SPSS missing data handling protocols.

### Sample size

Prior feasibility studies in non-veteran populations have reported sample sizes ranging from 32 to 49 patients [13]. Given the available cohort size and matched design, a matched sample size of 175 patients was considered sufficient to evaluate the study’s primary endpoints.

## Results

### Patient demographics

The entire cohort consisted of 602 patients undergoing elective umbilical hernia repair under GA (*n* = 427) or LA + MAC (*n* = 175). Of the patients included in the cohort, 93.7% were men, 70.1% were White, and 21.5% were actively smoking at the time of repair. The mean age was 55.7 ± 12.7 years, and the mean BMI was 32.1 ± 12.7 kg/m². The average size of the umbilical hernia neck was 2.2 ± 1.0 cm. Most patients had primary non-recurrent hernias (94%) at the time of the index operation, and 91.3% were repaired via suture rather than with mesh.

### GA vs. LA + MAC: unmatched cohort

Univariable analysis demonstrated that patients undergoing UHR via GA had higher BMI (32.4 ± 4.9 vs. 32.1 ± 4.5 Kg/m2; *p* = 0.01) and were more likely to have a prior (64.5% vs.54.6, *p* = 0.03) or current (23.5% vs.16.6, *p* = 0.06) history of smoking. Patients undergoing UHR under GA had more diabetes mellitus (34.7% vs. 24.6%; *p* = 0.02) and a lower serum albumin (4.0 ± 0.4 vs. 4.1 ± 0.3 mg/dL; *p* < 0.01). Additionally, patients undergoing UHR via GA had a larger hernia neck size (2.3 ± 1.1 vs. 2.2 ± 0.8 cm; *p* = 0.01). However, fewer patients undergoing GA presented with chronically incarcerated UH (27.4% vs. 48.0%; *p* < 0.01). Operative room time (skin-to-skin) was 3.1 min longer in the GA cohort compared to LA + MAC [*p* < 0.01; (Table 1)].

**Table 1:** Patient demographics and outcomes between unmatched and matched subjects. Further matching is depicted in Table 2

	Unmatched			Matched		
	General anesthesia (*n* = 427)	Local anesthesia (*n* = 175)	*p*-value	General anesthesia (*n* = 143)	Local anesthesia (*n* = 175)	*p*-value
Age (years-old ± SD)	55.8 ± 12.5	55.7 ± 13.3	0.76	51.88 ± 12.98	55.42 ± 13.25	0.02
BMI (Kg/m^2^ ± SD)	32.4 ± 4.9	32.1 ± 4.5	0.01	Matched see Table 2		
Male [n (%)]	403 (94.4)	161 (92.0)	0.3	130 (90.9)	161 (92.0)	0.73
White [n (%)]	301 (71.5)	117 (66.9)	0.26	94 (65.7)	117 (66.9)	0.83
Past history of smoking [n (%)]	273 (64.2)	95 (54.6)	0.03	Matched, see Table 2		
Current history of smoking [n (%)]	100 (23.5)	29 (16.6)	0.06			
Hypertension [n (%)]	279 (65.3)	101 (57.7)	0.08	80 (55.9)	101 (57.7)	0.75
Diabetes [n (%)]	148 (34.7)	43 (24.6)	0.02	Matched, see Table 2		
CKD [n (%)]	36 (8.4)	16 (9.1)	0.78	6 (4.2)	16 (9.1)	0.08
COPD [n (%)]	62 (14.5)	20 (11.5)	0.33	19 (13.3)	20 (11.4)	0.62
Cardiac [n (%)]	101 (23.7)	36 (20.6)	0.41	24 (16.8)	36 (20.6)	0.39
History of solid tumor [n (%)]	50 (17.6)	14 (10.9)	0.09	6 (4.2)	16 (9.1)	0.15
History of leukemia/lymphoma [n (%)]	7 (1.6)	2 (1.1)	0.95	2 (1.4)	4 (2.3)	0.68
History of liver dysfunction [n (%)]	55 (12.9)	19 (10.9)	0.49	10 (7.0)	19 (10.9)	0.23
Serum albumin [mg/dL (mean ± SD)	4.0 ± 0.4	4.1 ± 0.3	< 0.01	Matched, see Table 2		
Ascites [ n (%)]	12 (2.9)	1 (0.6)	0.09	1 (0.7)	1 (0.6)	0.89
ASA class I & II [n (%)]	165 (38.6)	69 (39.4)	0.85	70 (49)	69 (39.4)	0.09
ASA class III & IV [n (%)]	262 (61.4)	106 (60.6)		73 (51)	106 (60.6)	
History of chronic anticoagulation [n (%)]	40 (9.4)	10 (5.7)	0.14	7 (4.9)	10 (5.7)	0.75
Primary repair vs. mesh [n (%)]	50 (11.8)	8 (4.6)	0.01	Matched see Table 2		
Hernia size [cm (mean ± SD]	2.3 ± 1.1	2.2 ± 0.8	0.01	Matched see Table 2		
Chronic incarceration [n (%)]	117 (27.4)	84 (48.0)	< 0.01	Matched see Table 2		
Recurrent [n (%)]		2 (1.1)		Matched see Table 2		
OR time [min (mean ± SD)]	50.2 ± 25.7	47.1 ± 10.2	< 0.01	Matched see Table 2		
Recurrence [ n (%)]	20 (4.7)	2 (1.1)	0.04	4 (2.8)	2 (1.1)	0.3
Overall complications [n (%)]	54 (12.6)	4 (2.3)	< 0.01	14 (9.8)	4 (2.3)	< 0.01

*BMI* body mass index, *CKD* chronic kidney disease, *COPD* chronic obstructive pulmonary disease, *ASA* American Society of Anesthesia, *OR* operating room, *SD* standard deviation

### GA vs. LA + MAC: matched cohort

Propensity score matched analysis was undertaken to match 143 patients undergoing GA to 175 patients undergoing LA + MAC. After matching for BMI, history of smoking, recurrent repair, serum albumin, primary vs. mesh repair, hernia size, and operative room time, there were no statistically significant differences for all variables except for chronic incarceration, which was more common in patients undergoing LA + MAC (Table 2).

**Table 2:** Variables were matched between patients undergoing general vs. local anesthesia

	Matched		
	General anesthesia (n=143)	Local anesthesia(n=175)	*p*-value
BMI			
< 29.9 [(Kg/m2); n (%)]	45 (31.5)	61 (34.9)	0.52
> 30.0 [(Kg/m2); n (%)]	98 (68.5)	114 (65.1)	
Past history of smoking [n (%)]	80 (55.9)	95 (54.3)	0.77
Current history of smoking [n (%)]	25 (17.5)	29 (16.6)	0.83
Diabetes [n (%)]	31 (21.7)	43 (24.6)	0.54
Chronic incarceration (n (%)]	47 (32.9)	84 (48.0)	< 0.01
Recurrent repair [n (%)]	7 (4.9)	2 (1.1)	0.05
Serum albumin mg/dL			
≤ 3.4 [n (%)]	0 (0.0)	4 (2.4)	0.67
> 3.5 [n (%)]	143 (100.0)	168 (96)	
Primary repair vs. mesh			
Primary repair [n (%)]	137 (95.8)	167 (95.4)	0.87
Mesh [n (%)]	6 (4.2)	8 (4.6)	
Hernia size			
0.1 to 1.0 [cm, n (%)]	31 (21.7)	45 (25.7)	0.85
1.1 to 2.0 [cm, n (%)]	74 (51.7)	85 (48.6)	
2.1 to 3.0 [cm, n(%)]	34 (23.8)	41 (23.4)	
> 3.1 [cm, n (%)]	4 (2.8)	4 (2.3)	
OR time			
< 41.0 [min, n (%)]	119 (83.2)	148 (84.6)	0.74
> 41.0 [min, n (%)]	24 (16.8)	27 (15.4)	
Recurrence [n (%)]	4 (2.8)	2 (1.1)	0.3
Overall complications [n (%)]	14 (9.8)	4 (2.3)	< 0.01

### Recurrence

Recurrences were reported for the last known visit with an average follow-up of (9.5 ± 5.4 years). Recurrence was substantially higher in patients undergoing GA vs. LA + MAC in the unmatched cohort (4.7% vs. 1.1%; *p* < 0.01). However, after all variables were matched, this statistical significance dissipated (2.8% vs. 1.1%; *p* = 0.3) [Tables 1 and 2]. Time to recurrence was 4.7 ± 3.8 vs. 1.4 ± 1.2 years (*p* = 0.24) between patients undergoing UHR via GA vs. LA + MAC, respectively. Univariable analysis demonstrated no differences between patient undergoing GA vs. LA + MAC in recurrence rates with respect to age, BMI, hernia size, serum albumin level, primary repair vs. mesh or a history of current tobacco use (Table 3).

**Table 3:** Recurrence rates between subjects undergoing GA vs. LA + MAC

Recurrence	General (*n* = 20)	LA + MAC (*n* = 2)	*p*-value
Time to recurrence (years ± SD)	4.7 ± 3.8	1.4 ± 1.2	0.24
Age (years-old ± SD)	53.7 ± 12.3	52 ± 9.9	0.84
BMI (Kg/m^2^ ± SD)	33.3 ± 4.6	27.3 ± 7.2	0.11
Hernia size (cm ± SD)	3.4 ± 2.0	1.8 ± 0.4	0.26
Albumin (mg/dL ± SD)	4.0 ± 0.5	4.0 ± 0.5	0.26
Primary repair [n (%)]	19 (95.0)	2 (100)	0.74
Current Smoker [n (%)]	6 (30)	0 (0.0)	0.23

### Complications

Apart from recurrence, a list of postoperative complications is presented in Table 4. In total, 58 complications occurred in the entire cohort, 54 (GA) and 4 (LA + MAC). The most common complication was surgical site infection (SSI) in both cohorts. SSI was the only complication in patients undergoing (LA + MAC). No complication related to constipation or urinary retention was observed in patients undergoing LA + MAC (Table 4). Univariable analysis of patients with complications in each group showed no significant differences with regards to age, BMI, hernia size, serum albumin level, primary repair, or current history of tobacco use (Table 5), though this analysis is limited to 4 patients in the LA + MAC group.

**Table 4:** Overall complications in patients undergoing GA and LA + MAC

Overall complications	*n*	General	LA + MAC
Bleeding	1	0	0
Cellulitis	10	10	0
Constipation	5	5	0
Hematoma	2	2	0
Ileus	1	1	0
Nausea	3	3	0
Postop pain	6	6	0
Seroma	9	9	0
SSI	14	10	4
Urinary Retention	6	6	0
UTI	1	1	0

*SSI *surgical site infection*, UTI* urinary tract infection, *LA* local anesthesia, MAC monitored anesthesia care in the unmatched cohort

**Table 5:** Demographic differences between subjects who had complications under GA vs. LA + MAC in the unmatched cohort

Overall complications	General (*n* = 54)	LA + MAC (*n* = 4)	*p*-value
Age (years-old ± SD)	57.8 ± 11.2	63.0 ± 6.2	0.37
BMI (Kg/m^2^ ± SD)	32.8 ± 4.1	32.3 ± 2.1	0.8
Hernia size (cm ± SD)	2.7 ± 1.5	2.1 ± 1.1	0.46
Albumin (mg/dL ± SD)	4.0 ± 0.6	4.1 ± 0.3	0.97
Primary repair [n (%)]	12 (81.5)	3 (75.0)	0.9
Current Smoker [n (%)]	10 (18.5)	2 (50.0)	0.14

In the unmatched cohort, the rate of complications between GA vs. LA + MAC was 12.6% vs. 2.3%; *p* < 0.01). Following correction by PSMA, this statistic significance remained (9.8% vs. 2.3%; *p* < 0.01) (Tables 1 and 2).

### Operative room times

All times were longer in the GA vs. LA + MAC cohorts: from the time the patient arrived in the operating room to the start of the operation (25.5 ± 11.9 vs. 24.2 ± 8.5; *p* < 0.01); skin-to-skin time 50.2 ± 25.7 vs. 47.1 ± 10.2; *p* < 0.01); close skin to out of the OR (9.6 ± 6.9 vs. 8.8 ± 3.1 ; *p* < 0.01), and total OR times (137.1 ± 57.6 vs. 129.±26.1; *p* < 0.01). In all, 5.3 min were saved in the LA + MAC cohort. The average time that patients spend in the post-operative acute unit (PACU) is 50 min, compared to zero in the LA + MAC group.

## Discussion

Evidence regarding outcomes for UHR under LA + MAC is scarce in the literature. A previous systematic review evaluated the safety of LA for UHR with respect to duration of surgery, SSI, perioperative and postoperative complications, postoperative pain, hernia recurrence, time before discharge, as well as patient satisfaction. This analysis included nine studies from 2000 to 2013. Papers that were published in languages other than Dutch, English, or German were excluded. Six studies were prospective, and three retrospective. No RCTs were found in this review. The overall level of evidence was graded as 3B.

The surgical and anesthesia techniques were highly variable among studies. Similarly, outcomes were reported differently. Study sample size ranged from 32 to 326. UHR performed under LA was associated with shorter operative time, and 90% of patients were discharged within 24 h compared to 47% undergoing GA. Overall patient satisfaction was 89% to 97%. Complications reported included SSI (1% to 12.9%), seromas (3% to 8.9%), and hematomas (1%). But there were no comparisons between the complication and the type of anesthesia. Recurrence rates were reported in four articles (2% to 7.4%), but no statistical difference by anesthesia type was reported in any of the studies. Other than reduced operative time, decreased hospital length of stay, and safety of LA, the authors concluded that no consensus could be generated due to the variability of the studies and suggested a RCT to determine the feasibility of LA for UHR [6].

A more recent systematic review evaluated ventral hernia repairs (< 5 cm) under LA. Studies published between 1966 and 2023 were included and consisted of 33 manuscripts. Hernia-related complications, length of hospital stay, and cost were the main outcomes evaluated in this review. This study concluded that complications were low with LA [wound infections and hematomas (0.3% to 2.0%)], and showed that there was early mobilization in patients receiving LA. Further, in patients receiving LA, postoperative pain was minimal, and patient satisfaction was 90–97%. The authors supported the use of LA for ventral hernias less than 5.o cm [7].

However, this paper had a wide range of abdominal wall hernias: ventral hernias (*n* = 8), umbilical (*n* = 6), incisional (*n* = 4), Spigelian (*n* = 4), combination of inguinal, femoral, and umbilical (*n* = 3), paraumbilical (*n* = 3), and others (*n* = 5). The type and volume of LA were highly variable between studies. Complications were also reported differently in all the papers. Recurrence rate was 0.3% to 2.5%, which was similar for patients undergoing both LA and GA. The main conclusion of this study was that LA was feasible, safe, and had good patient satisfaction in patients undergoing ventral hernia repair [7].

Data from the Veterans Affairs Surgical Quality Improvement Program (VASQIP) further supports the potential benefits for LA. In frail Veteran Patients (risk analysis index ≥ 30) UHR performed under LA (*n* = 167) vs. GA (*n* = 736) was associated with shorter operative times (approximately 9 min faster; *p* < 0.0001) and significantly lower 30-day complication rates (1.6% vs. 2.5%; *p* < 0.0001). This study also assessed potential cost savings associated with LA [8]. There were no differences in outcomes when the entire cohort of patients receiving LA vs. GA was compared; it was only for frail Veterans that the difference was observed. A potential saving for the VA health care system was attributed to the faster OR and recovery times [8].

A prior feasibility study (*n* = 53) conducted at the VA North Texas Health Care System evaluated Veteran patients undergoing elective UHR under LA + MAC compared with GA. Patients in the LA + MAC cohort demonstrated similar 30-day postoperative complication rates despite having a higher American Society of Anesthesiologists (ASA) class and a greater burden of cardiovascular comorbidity. On univariable analysis, LA + MAC was associated with reduced operative room utilization, with an approximate 50-minute time savings, largely attributable to the avoidance of post-anesthesia care unit (PACU) recovery time [1].

To date, no controlled studies have directly compared GA to LA with or without MAC for UHR. We undertook this analysis to determine if LA + MAC would have better outcomes compared to GA while controlling key differences, including the same surgeon, similar patient population, similar BMI, history of smoking, diabetes, history of recurrent hernia, serum albumin, primary vs. mesh repair, hernia size, and operative room time. A PSMA model was utilized for the analysis as previously described [9]. This study represents one of the largest single-surgeon analyses comparing GA and LA + MAC for elective adult open UHR and the first to apply propensity matching in this setting.

The present analysis showed that after correcting for major variables, patients undergoing UHR via LA + MAC had similar recurrence rates (1.1% vs. 2.8%; *p* = 0.3). This recurrence rate compares favorably with previously reported systematic reviews [6, 7]. Apart from recurrence, the entire cohort experienced an overall complication rate of 9.6%, which also compares favorably with previous reports in similar cohorts. However, in this study, there was a substantial reduction in overall complications in patients receiving LA + MAC compared to GA (2.3% vs. 9.8%). In agreement with other studies, we found that the most common post-operative complications were SSI, cellulitis, and hematomas. Adverse outcomes related to GA (constipation, urinary retention, ileus, and nausea) did not occur in the LA + MAC cohort. Only four SSIs were observed in LA + MAC patients.

The marked reduction in complications is notable. Complications such as urinary retention, ileus, and nausea were absent in the LA + MAC cohort. Additionally, surgical site infections were substantially lower. This may relate to physiologic differences associated with GA, including hypothermia, fluid shifts, and metabolic perturbations [10].

After adjustment for confounders, UHR under LA + MAC was associated with a modest reduction in operative time (5.3 min). Together with the approximately 50 min saved by eliminating PACU utilization, this likely represents a meaningful reduction in healthcare resource utilization and cost [8].

The benefits of LA over GA have been well established in inguinal hernia repair, where reduced complications and costs have been consistently demonstrated [11]. Our findings extend these observations to UHR. Despite this, the adoption of LA for hernia repair remains limited. Only 7% of surgeons reportedly utilize LA for open inguinal hernia repair [12], and VASQIP data indicate that only 13.4% of UHRs are performed under LA compared to 86.7% under GA [8]. This discrepancy likely reflects surgeon preference rather than evidence-based practice [13]. We hope that the report of this study, along with the data from inguinal hernia repair under LA + MAC, invites surgeons to adopt this modality over GA, especially in frail patients.

This study has several limitations. It is a retrospective review of a prospectively maintained database. Thus, retrieving data in this fashion is never as accurate as data collection in RCTs. Our study involves the repair of hernias, mostly via primary repair (91%), even in hernias with a large hernia neck (> 2.0 cm). This has been an acceptable practice in our patient population [3–5]. However, RCTs, meta-analyses, and society guidelines recommend mesh placement for most UHs > 1.0 cm to prevent recurrence. We do not contest these recommendations, but for our patient population, tissue repair has been feasible. Thus, the results of our study might not apply uniformly to other cohorts. Further, LA + MAC was implemented in the last seven years of a 21-year surgical practice repairing UHs. Thus, a temporal variation in higher skills in the repair of UHRs is possible. We have made efforts to match both groups in terms of all key variables. Despite these efforts, while statistical significance was comparable, numerical differences remained and they might be clinically important. However, even with all these limitations, the main goal was to compare LA + MAC vs. GA while controlling for key variables associated with outcomes following UHR. But nothing replaces data generated from RCTs. In the absence of an RCT, PSMA remains the best available evidence for UHRs under LA + MAC. We hope this work serves as a foundation to encourage the development of prospective randomized trials in this field.

## Conclusions

The current evidence supporting umbilical hernia repair (UHR) under local anesthesia (LA) derives from two systematic reviews characterized by substantial heterogeneity, limiting conclusions primarily to the feasibility and safety of LA with monitored anesthesia care (MAC) for UHR. At present, no level I evidence compares LA + MAC with general anesthesia (GA) for patients undergoing UHR. In the present study, we evaluated outcomes among Veteran patients undergoing UHR with LA + MAC versus GA using propensity-score–matched analysis. After adjustment for major variables influencing UHR outcomes, recurrence rates were similar between the two groups. However, patients undergoing UHR under LA + MAC experienced a significantly lower overall complication rate. In addition, the LA + MAC cohort had a moderate reduction in operating room time. The lower complication rate, shorter operative time, and avoidance of post-anesthesia care unit utilization may together translate into meaningful reductions in health care costs in this population. These findings support broader adoption of LA + MAC for UHR, although randomized controlled trials are still needed.
